# Free-living wrist and hip accelerometry forecast cognitive decline among older adults without dementia over 1- or 5-years in two distinct observational cohorts

**DOI:** 10.1038/s41514-022-00087-w

**Published:** 2022-06-06

**Authors:** Chengjian Shi, Niser Babiker, Jacek K. Urbanek, Robert L. Grossman, Megan Huisingh-Scheetz, Andrey Rzhetsky

**Affiliations:** 1 Pritzker School for Molecular Engineering, University of Chicago, Chicago, IL 60637 USA; 2 Department of Medicine, University of Chicago, Chicago, IL 60637 USA; 3 Department of Medicine, Johns Hopkins University, Baltimore, MD 21287 USA; 4 Department of Computer Science, University of Chicago, Chicago, IL 60637 USA; 5 Department of Human Genetics, University of Chicago, Chicago, IL 60637 USA

**Keywords:** Epidemiology, Geriatrics

## Abstract

The prevalence of major neurocognitive disorders is expected to rise over the next 3 decades as the number of adults **≥**65 years old increases. Noninvasive screening capable of flagging individuals most at risk of subsequent cognitive decline could trigger closer monitoring and preventive strategies. In this study, we used free-living accelerometry data to forecast cognitive decline within 1- or 5-years in older adults without dementia using two cohorts. The first cohort, recruited in the south side of Chicago, wore hip accelerometers for 7 continuous days. The second cohort, nationally recruited, wore wrist accelerometers continuously for 72 h. Separate classifier models forecasted 1-year cognitive decline with over 85% accuracy using hip data and forecasted 5-year cognitive decline with nearly 70% accuracy using wrist data, significant improvements compared to demographics and comorbidities alone. The proposed models are readily translatable to clinical practices serving ageing populations.

## Introduction

Alzheimer’s disease and related major neurocognitive disorders (ADRD) affect over 50 million people worldwide, with an increase of 10 million new cases per year^1^. The ADRD disease burden is expected to increase as the world population ages^2,3^. ADRD disproportionately affects socioeconomically disadvantaged groups and minorities^4^ and is associated with lower quality of life, increased mortality, care dependence, and institutionalization. Preservation of cognitive abilities and a positive mindset may maintain quality of life in later years^5^. Few US Food and Drug Administration (FDA) approved treatment options exist at this time; therefore, the mainstay of current management remains on the prevention side^6^. Cognitive trajectories vary widely among older adults, with recent studies showing that different races experience varying rates of decline^7,8^. Finding sensitive forecasters of early decline could trigger more frequent monitoring and aggressive preventative interventions, advance care planning, and even ADRD research study eligibility^9^.

There is an acute need for easily deployed, noninvasive, clinical tools to identify cognitively intact older adults most at risk of subsequent cognitive decline. Certain clinical and environmental factors including age, gender, education, body mass index, neighborhood socioeconomic status, and history of stroke or diabetes are easy to gather clinically during a visit or even during a telephone screen^10^. In a meta-analysis, structural and functional aspects of one’s social environment (including network size, social activity, and loneliness) are also predictive of cognitive decline among older adults^11^. Genetic susceptibilities, such as APOE carrier status, can improve forecast models but are more invasive for patients to collect^12^.

Wearable sensors have been gaining attention for their ability to remotely collect free-living activity and sleep patterns and the association of these patterns with other important age-related conditions: frailty^13–15^, disability^16^, social disengagement^17^, and death^18^. The relationship between free-living activity and cognitive performance has been less studied. In cross-section, greater activity volume (highest and middle tertiles of active minutes/day) was associated with better processing speed among cognitively intact adults at risk of mobility disability^19^ and steps/day were associated with better executive functioning in healthy older adults^20^. Longitudinally, cognitively intact older adults with a higher percentage of moderate to vigorous physical activity (MVPA) per week had a lower risk of cognitive impairment and better maintenance of executive function and memory over an average of 3 years^21^. However, these findings were not consistent across racial/ethnic groups. A higher percent of MVPA predicted maintenance of only memory and not executive function in African American/Black adults, as compared to White adults^21^.

Few prior studies have leveraged the high-resolution nature of accelerometer data in analyses to maximize unique pattern recognition that may differentiate health risk across individuals, a concept familiar to those studying precision medicine. While accelerometry is not currently used in routine clinical care, it has been increasingly used in major research studies to remotely assess older adult health and poses significant advantages in the era of telehealth^22–28^. Translation of accelerometry in clinical practice has been challenged by the lack of accelerometry tools with clear clinical applications and the inability to apply research findings across device body locations and manufacturers.

The objective of this study was to significantly advance the prior work on forecasting early cognitive decline among older adults without dementia by discovering prognostic, free-living accelerometry patterns using 24-h data. We considered 98 accelerometry measures, the most comprehensive set of movement-related measures in a study of its kind to date. With a screening clinical application in mind, we chose a simple, binary clinical outcome that is most relevant to triggering clinical or research decision making: any cognitive decline versus stable or improving cognition. We further probed into the generalizability of the developed methodology, by applying it to data from two studies that gathered data from two different accelerometers worn at different body locations and with different wear protocols.

## Results

### Cohort characteristics

The characteristics of the two study cohorts are shown in Table 1. The hip accelerometry cohort was older (mean age 73.2), had a slightly higher baseline Montreal Cognitive Assessment (MoCA) score (mean 25.4), and included a larger proportion of females (80.9%) and those self-identifying as African American (81.7%) than the wrist accelerometry cohort (mean age 70.0, mean MoCA 23.4, proportion female 59.1%, proportion African American/Black 11.3%).

**Table 1:** Demographic composition of the two accelerometry cohorts.

Characteristics	Hip accelerometry cohort (*N* = 115)	Wrist accelerometry cohort (*N* = 575)
Age (year)	73.2 (5.9)	67.0 (7.9)
Gender (female)	93 (80.9 %)	340 (59.1 %)
Race		
African American/Black	94 (81.7 %)	65 (11.3 %)
White	21 (18.3 %)	423 (73.6 %)
Hispanic	None	67 (11.7 %)
Other	None	20 (3.5 %)
Education		
Some college or junior college	45 (31.9 %)	211 (36.7 %)
Post-graduate	28 (24.4 %)	137 (23.8 %)
College graduate	27 (23.5 %)	
High School graduate or GED (grade 12)	11 (9.6 %)	135 (23.5 %)
Some high school (grades 9–11)	4 (3.5 %)	92 (16.0 %)
Income ($/month)		
*<*2000	65 (50.0 %)	-
2000–3999	40 (30.8 %)	-
4000–5999	16 (12.3 %)	-
≥6000	9 (6.9%)	-
Charlson Comorbidity Index Score	1 (1.3)	0.9 (1.2)
MoCA (baseline)	25.4 (2.6)	23.4 (4.0)
MoCA		
(Hip: 1 year; Wrist: 5 years)	25.6 (3.0)	22.6 (4.4)

### Demographic and clinical predictors of cognitive decline

As we observe in Table 2, the clinical characteristics had somewhat limited capability to distinguish between those with stable/improving cognition versus those with declining cognition at 1 and 5 years in the local and national cohorts, respectively. We provided a full dictionary of features in the Supplemental Data.

**Table 2:** Effect size of predictors of 1- and 5-year cognitive function, the values shown for each feature are Cohen’s D/odds ratio with 95% confidence intervals.

Characteristics	∆_+_ Vs ∆_−_	∆_+_ Vs ∆_−_
	Hip-worn (*N* = 115)	Wrist-worn (*N* = 575)
Age (year)^a^	0.457 (0.103, 0.801)	0.284 (0.119, 0.449)
Gender (female)^b^	3.533 (1.223, 10.207)	0.865 (0.620, 1.207)
Race^b^		
African American/Black	1.124 (0.472, 2.680)	0.897 (0.741, 1.086)
White	-	1.014 (0.795, 1.293)
Hispanic	-	1.143 (0.873, 1.499)
Other	-	1.344 (0.769, 2.345)
Education^b^		
Some college or junior college	2.265 (1.092, 4.697)	0.850 (0.605, 1.195)
Post-graduate	0.973 (0.443, 2.134)	0.909 (0.619, 1.335)
College graduate	0.532 (0.233, 1.215)	
High School graduate or GED	0.437 (0.110, 1.726)	1.018 (0.652, 1.591)
Some high school (grades 9–11)	0.821 (0.132, 5.088)	1.337 (0.908, 1.969)
Income ($/month)^b^		
*<*2000	1.283 (0.641, 2.566)	-
2000–3999	0.656 (0.306, 1.405)	-
4000–5999	0.715 (0.244, 2.101)	-
≥6000	2.653 (0.634, 11.110)	-
Charlson Comorbidity Index Score^a^	0.135 (−0.213, 0.485)	−0.008 (−0.172, 0.156)
MoCA (baseline)^a^	0.572 (0.216, 0.928)	0.418 (0.252, 0.584)

^a^Effect size computed by Cohen’s D method.

^b^Effect size computed by odds ratio.

### Combining demographic, clinical, and accelerometry predictors of cognitive decline

To investigate the importance of the accelerometry activity measures and harmonic features beyond that of the demographic and clinical characteristics on cognitive degradation forecasting, we trained CDPred on three different sets of measures: (1) the CDPred basic model using demographic and clinical characteristics; (2) the CDPred-4 model using demographic and clinical characteristics with C4 and V4; (3) the CDPred-4+ model using demographic and clinical characteristics, C4 and V4, plus the harmonic features derived from accelerometry. The number of features in each model are listed in Table 3. To summarize, we compared three models: CDPred, CDPred-4, and CDPred-4+. CDPred includes the baseline demographic and clinical features. CDPred-4 model uses the baseline demographic and clinical features and two baseline accelerometry metrics (C4 and V4). CDPred-4+ models use the full gamut of information: the baseline demographic and clinical features, the two baseline accelerometry metrics, and all extracted 98 accelerometry harmonic features.

**Table 3:** Number of predictors in the three hip and wrist accelerometry models.

Model	Hip-worn	Wrist-worn
CDPred	7	6
CDPred-4	9	8
CDPred-4+	105	104

### Performance of the models

**Fig. 1:**
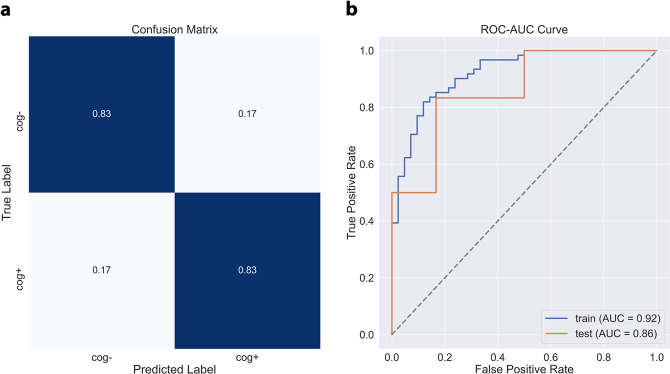
Confusion matrix and ROC-AUC curve summarizing experiments with data recorded for the hip accelerometry cohort. The figure shows **a** confusion matrix and **b** ROC-AUC curve for the held-out sample.

**Fig. 2:**
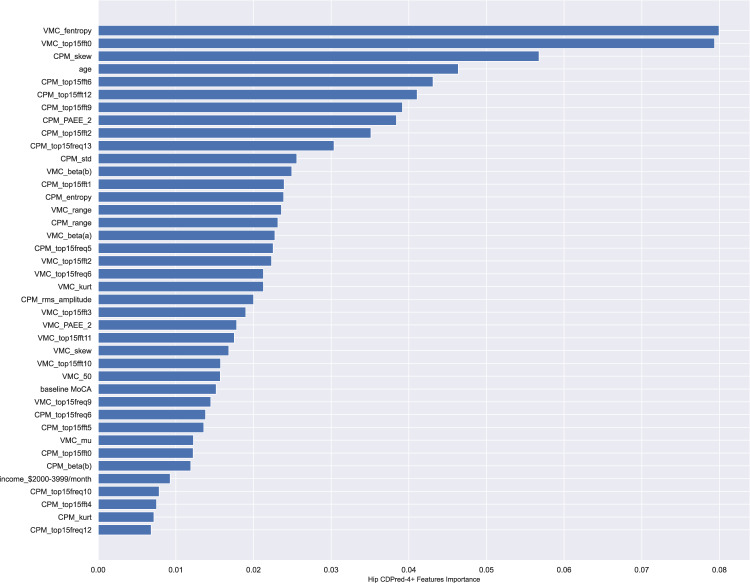
Relative importance of predictive features in CDPred-4+ experiments with the hip accelerometry cohort. The features are listed in the order of decreasing importance, from top to bottom of the graph.

**Fig. 3:**
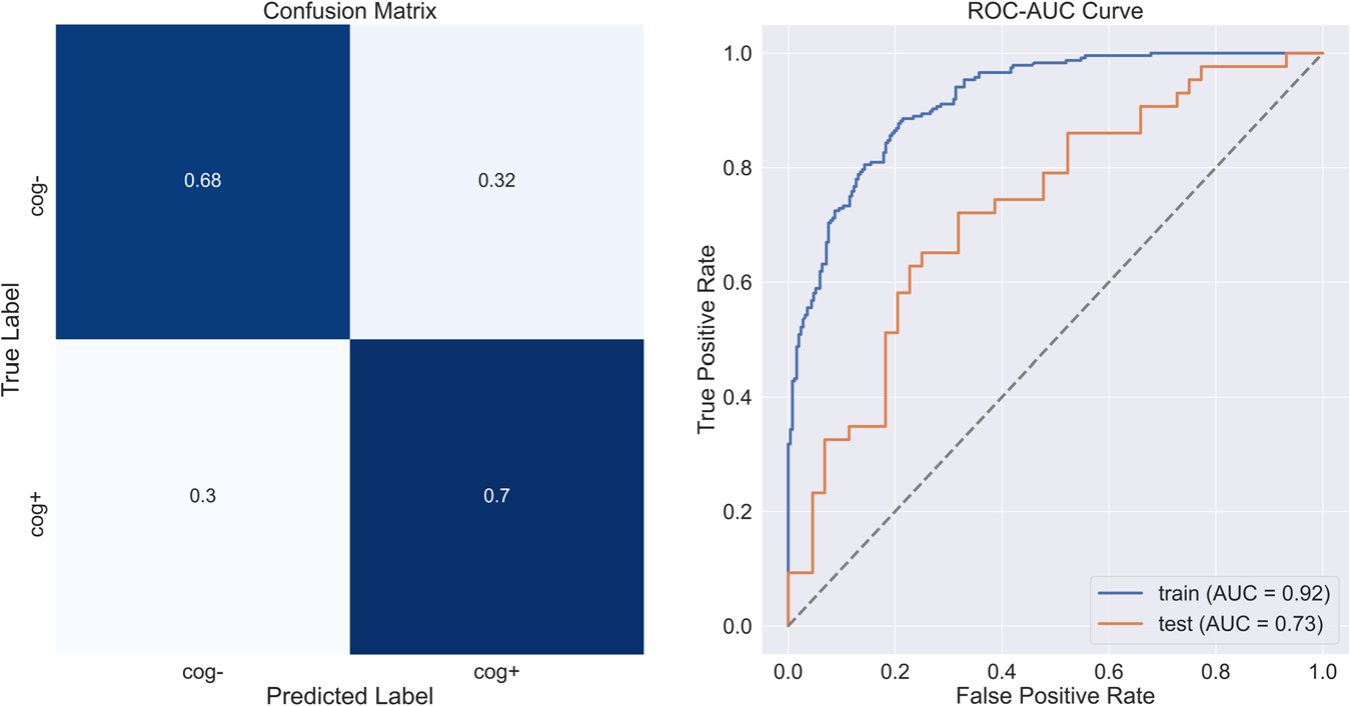
Confusion matrix and ROC-AUC curve summarizing experiments with data recorded for the wrist accelerometry cohort. The figure shows **a** confusion matrix and **b** ROC-AUC curve for the held-out sample.

**Fig. 4:**
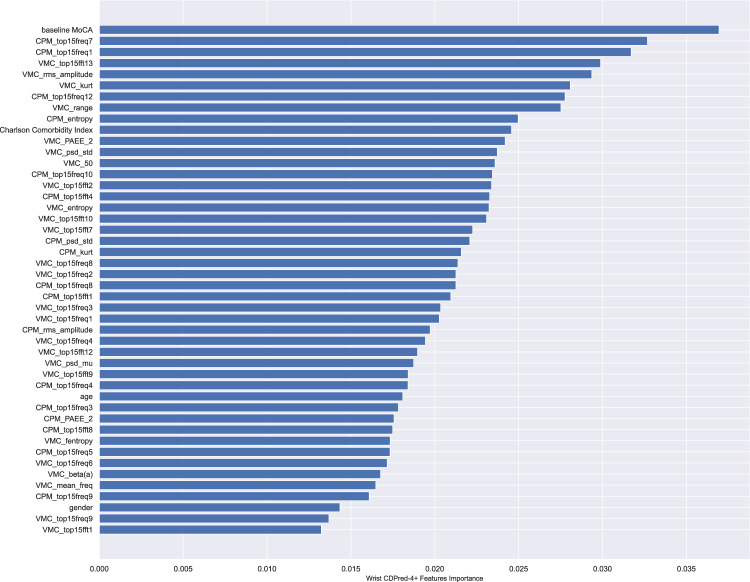
Relative importance of predictive features in CDPred-4+ experiments with the wrist accelerometry cohort. The features are listed in the order of decreasing importance, from top to bottom of the graph.

The model performance metrics on the hold-out samples are shown in Table 4. The CDPred-4+ model including all measures predicted cognitive decline 1 year later with an accuracy of over 85% (hip accelerometry cohort) and predicted cognitive decline 5 years later with nearly 70% accuracy (wrist accelerometry cohort). The hip-worn accelerometry confusion matrix and ROC-AUC for the CDPred-4+ model in the hold-out sample is shown in Fig. 1. Figure 2 shows predictors sorted by relative importance, from the highest to lowest, excluding features with zero importance. Similarly, we show the confusion matrix and ROC-AUC for the CDPred-4+ model in the wrist-worn accelerometer data in Fig. 3, and nonzero predictor relative importance sorted in descending order in Fig. 4.

**Table 4:** Predicting cognitive decline: performance of classifier models with an increasing number of predictors.

Model	Accuracy	AUC (std^a^)
CDPred (hip device)	0.75	0.74 (0.11)
CDPred-4 (hip device)	0.78	0.75 (0.10)
CDPred-4+ (hip device)	0.84	0.86 (0.11)
CDPred (wrist device)	0.66	0.65 (0.05)
CDPred-4 (wrist device)	0.67	0.66 (0.05)
CDPred-4+ (wrist device)	0.69	0.73 (0.05)

^a^Standard deviation.

## Discussion

Our model significantly expands work previously published in this space. Casanova et al. (2020) similarly used a Random Forest Classifier to distinguish cognitive trajectories^12^. Three classes, low-, medium-, and high-risk trajectories were created using a combination of baseline and repeated cognitive performance scores. This study found that age, gender, education, BMI, stroke, diabetes, neighborhood socioeconomic status, and APOE carrier status were among the top predictors of cognitive trajectories. They did not include accelerometry assessments. We found that the accelerometry pattern features outperformed many demographic and clinical characteristics in predicting cognitive decline in a community-dwelling cohort, suggesting the potential value of noninvasive and remote accelerometry in augmenting the clinical evaluation.

Our analyses have shown that, compared to simpler, clinical models predicting cognitive decline (e.g., using only demographic and clinical characteristics), our accelerometry-based classifier model performs significantly better. This model uniquely identifies preclinical cognitive decline among older adults without a diagnosis of dementia over short (1-year) and longer-term (5-year) follow-up. The model was robust to varying wear protocols (7 days versus 72 h), device location (hip versus wrist), and device manufacturer. In both models, many accelerometry features were rated more ‘important’ in distinguishing those who experienced any decline in cognition than many demographic and clinical characteristics including age. We are hopeful that the current level of model performance may be useful to flag older adults most vulnerable to subsequent cognitive decline. We note and emphasize that accelerometry currently has no diagnostic capacity for any clinical diseases; its role in the current study is restricted to an assessment of day-to-day movement (accelerations and decelerations) which seems to reflect some level of health, here cognitive, risk.

### Limitations

This study has several limitations worth mentioning. It is not technically possible to guarantee (or test) that there was no overlap between the two cohorts used in this study. The NSHAP dataset did collect zip code information on participants, but the FACE Aging dataset did not collect any address information. Since the FACE Aging dataset is composed of study participants residing in the few neighborhoods surrounding the University of Chicago and NSHAP sampled across the nation using a complex sampling design based on census tracts, if overlap occurred, it would have been a very small number of participants.

Another limitation of the current study is, despite the importance of understanding ADRD for socio-demographically disadvantaged groups, the datasets for this study were not sufficient in size for understanding the relative predictive power of the models for different sociodemographic groups. We did include effect size measures for different race/ethnicities in Table 2 and the effect size of accelerometer features on race/ethnicity in Supplementary Table 1 as the first step in this direction. We show ranked effect sizes of individual features in Supplemental Fig. 1.

Our forecast model was only 70–80% accurate leaving room for improvement. It is likely that our forecast model could be enhanced in future work to reach higher and more consistent accuracy. This can be achieved by including additional metrics derived from accelerometry data, possibly using additional physiologic sensors such as heart rate monitoring to capture richer data, and incorporating additional clinical data, such as blood or genetic markers, family history of dementia, and current medications.

The wrist accelerometry model did not perform as well as the hip accelerometry model. The weaker performance of the wrist accelerometry location might be due to the shorter wear protocols, increased motion “noise” related to the position, and longer follow-up cognitive assessments. Future work comparing more similar wear protocols and devices, even if worn at different body locations, would be of value.

Our experiments show that this predictive model can forecast preclinical cognitive decline using data from dissimilar accelerometry device locations, wear protocols, follow-up times, and unique cohorts. Hip- and wrist-worn accelerometers are subject to unique patterns of movements in space, yet data from both accelerometry devices improved the predictive capacity of the respective models. The somewhat inferior performance of the wrist accelerometry, among other factors, may be related to the shorter wear protocol (72 h versus 7 days versus “noisy” data at the wrist) and longer follow-up (5 years). A major challenge to accelerometry research and clinical translation has been the reliance on a particular device location, protocol duration, and/or proprietary data processing software for generating accelerometry measures^29,30^. These limitations have stimulated movement toward using open source programs or approaches for generating accelerometry metrics, as we have done in this study, and identifying methodologic approaches applicable across multiple devices and varying wear protocols.

## Methods

### Study populations

To evaluate the robustness of our proposed methodology, we used information about two non-overlapping cohorts of community-dwelling older adults, one cohort equipped with hip-based and another with wrist-based accelerometers.

#### Hip accelerometry cohort: frailty, aging, body composition and energy expenditure in aging (FACE aging) study

Study participants (*n* = 151) were recruited from the community around the primary geriatrics practice site for the University of Chicago located on the south side of Chicago. The sample was limited to community-dwelling (not living in residential care) older adults, 65 or older. Exclusion criteria included hospitalization, surgery, or procedure within 2 months of participating in the study; addition or change in dose of the thyroid (e.g, levothyroxine) or a diuretic (e.g, furosemide, hydrochlorothiazide, or spironolactone) medication within 2 months of participating in the study; use of oral steroids; use of beta-blockers (e.g., metoprolol, atenolol, or carvedilol); persistent hyperglycemia greater than 250; life expectancy less than 1 year; and history of moderate or advanced dementia or Montreal Cognitive Assessment (MoCA) less than or equal to 18. Hospital, surgery, medication, and hyperglycemia exclusion criteria were required to optimize resting metabolic rate testing at baseline (data not used in this analysis). Data collection occurred over multiple evaluations: (1) baseline survey and physical exam in the clinic, (2) a 7-day free-living hip accelerometry protocol immediately following the exam, (3) fasting resting metabolic rate measurement with indirect calorimetry and DEXA scan for body composition within 2 weeks of baseline assessment, (4) a 1-year follow-up survey and physical exam in the clinic. We restricted the study sample to participants with complete clinical data and one or more valid (≥10 daytime hours) accelerometer-wear days, which left us with 115 participants eligible for our classifier development.

##### Hip accelerometer protocol

Hip accelerometry data were collected from all participants at baseline. Following the baseline survey and physical exam, an Actigraph wGT3X+ hip accelerometer was placed over the participant’s mid, anterior right hip and secured with an elastic belt. Study participants were asked to keep the device on their hip continuously for 7 full days (including during bathing or showering). The accelerometers recorded data at a frequency of 30 Hz. The subsecond-level data were extracted from the devices using the ActiLife software (version 6.0). The low-frequency extension filter was NOT applied.

#### Wrist accelerometry cohort: the national social life, health, and aging project

We used wrist accelerometry data generated by the National Social Life, Health, and Aging Project (NSHAP) as the sample. NSHAP is a nationally-representative, longitudinal survey study that collects extensive information on physical, mental, cognitive, and social health in United Study, community-dwelling older adults^31^. The first wave of NSHAP was in 2005–6 which included a nationally, statistically representative sample of community-dwelling adults born between 1920–47 (aged 57–85) and over-sampled for African-Americans, Hispanics, and males; 3377 respondents participated (weighted response rate = 75.5%). Five years later (2010–11), respondents were re-interviewed as were their cohabiting spouse or partner, for a total *n* = 3377. Interviews were conducted in the homes of each respondent by professional interviewers from NORC at the University of Chicago. A random subset of the 2010–11 respondents were invited to participate in a wrist accelerometry protocol, the data used in the current analysis.

##### Wrist accelerometry sub-study protocol

Wrist accelerometry data were collected from a randomly selected subset of 793 respondents in the 2010–2011 data collection wave. The 2010–2011 accelerometry protocol has been previously described in detail^28^. Briefly, randomly selected respondents in the 2010–2011 data collection wave were asked to wear an ActiWatch Spectrum^®^ on their non-dominant wrist continuously for 72 consecutive hours (including during bathing or swimming activities)^28^. The accelerometers recorded data at a frequency of 32 Hz. Upon receiving returned devices, data were downloaded from the device and then pre-processed using the Actiware^®^ software^32^. The maximum absolute value was computed for each second; the sum of these absolute values was then computed for every 15-s epoch. The ActiWatch has a galvanic heat sensor that identifies when a device is on the wrist. All non-wear periods were excluded (only 0.17% of epochs across all wake data were classified as non-wear). Days with at least 10 h of daytime recording were considered “valid”; days with less than 10 h of daytime recording were excluded. The 24-h time interval was used to generate the wrist accelerometry features for this analysis. The study sample was restricted to participants with complete clinical data and ≥1 valid accelerometry wear day which left 584 participants eligible for our classifier development.

### Clinical measures

#### Cognitive function

##### Hip accelerometry cohort

The Montreal Cognitive Assessment (MoCA) was used to determine cognitive function at baseline and 1-year follow-up for the hip accelerometry training sample. The MoCA evaluates seven domains of cognitive function. The scale ranges from 0 to 30 with higher scores indicating better function. Because education was included as a covariate and our primary focus was on change in cognition, we did not add an additional point to the MoCA score for education levels below 12 years as is clinically done^33^.

##### Wrist accelerometry cohort

In the wrist accelerometry sample, cognitive function was assessed in 2010–11 and 2015–16 using the survey-adapted Montreal Cognitive Assessment (MoCA-SA) as previously described in detail^32^. MoCA scores (range 0–30) are estimated from the 18-item MoCA-SA using a linear prediction model^34,35^. The wrist accelerometer data were collected in 2010–11 along with a baseline MoCA. The MoCA was repeated in 2015–16.

In both cohorts, we calculated cognitive change as a difference in MoCA scores between the baseline and follow-up assessments (1 year for the hip accelerometry cohort and 5 years for the wrist accelerometry cohort):

1 $$\Delta = {\mathrm{MoCA}}_{{{{\mathrm{follow}}}} - {{{\mathrm{up}}}}}-{\mathrm{MoCA}}_{{{{\mathrm{baseline}}}}}.$$

Patients with deteriorating MoCA scores (∆ *<* 0) were assigned to the cognitively declined group, denoted as ∆_−_. The remaining patients were assigned to the group with a lack of cognitive decline, denoted as ∆_+_. The ratio of ∆_+_/∆_−_ was 67/48 in hip-worn- and 279/296 in wrist-worn-accelerometer cohorts. The range of 1-year cognitive change (hip) was −8 to 6. The range of 5-year cognitive change (wrist) was −14.9 to 14.9.

#### Covariates

The covariates were measured similarly across cohorts.

##### Hip accelerometry cohort

In the hip accelerometry cohort, age, race, gender (female vs. male), education (high school≥ vs. < high school graduate), and monthly income category ($0 < 2000, $2000–3999, $4000–5999, and $6000+) were recorded through self-reported measures. Options for the race included Black or African American and Other (White, American Indian or Alaska Native, Asian Indian, Chinese, Filipino, Japanese, Korean, Vietnamese, Other Asian, Native Hawaiian, Guamanian or Chamorro, Samoan, Other Pacific Islander, or Other). No participants reported Hispanic ethnicity. Information on previously diagnosed comorbidities (self-reported and chart review) was recorded and scored using the Charlson Comorbidity Index and included heart attack, asthma, emphysema, chronic bronchitis, a chronic obstructive pulmonary disorder, peripheral vascular disease, liver disease, diabetes, and cancer (continuous, range 0–30)^36^.

##### Wrist accelerometry cohort

In the wrist accelerometry cohort, age (centered, continuous) was calculated using the reported date of birth and interview date. Gender (female versus male), race (White/Caucasian, Black/African American, other), and Hispanic ethnicity^37^. A modified Charlson Comorbidity Index (range 0–16, continuous) was constructed using self-reported comorbidity data in the 2010–2011 data collection wave. Respondents were asked whether they had ever been told by a doctor that they had any of the following conditions (number of points given in parentheses): congestive heart failure (1), heart attack (1), coronary procedure (1), stroke (1), diabetes (1), rheumatoid arthritis (1), asthma, emphysema, chronic obstructive pulmonary disease, or chronic bronchitis (1), dementia (1), non-metastatic cancer excluding skin cancer (2), or metastatic cancer excluding skin cancer (6)^38^.

### Accelerometer data preparation

Data were restricted to enrollees with at least one valid day. We calculated the Euclidean norm minus one (ENMO), counts per minute (CPM), and vector magnitude count (VMC) for each participant using the hip and wrist data. To calculate these metrics, the accelerometry data needed to be in the form of the vector magnitude/Euclidean norm. The subsecond-level wrist accelerometry data were already converted to the vector magnitude/Euclidean norm by the manufacturer’s software, 1 data point for every 15-s epoch, where *N* = 24 h per day × 60 min per hour × 4 samples per minute = 5760 samples per day for wrist-worn accelerometer data.

The hip accelerometer data were in the form (*x*(*t*), *y*(*t*), z(*t*)), where *x*(*t*), *y*(*t*), and *z*(*t*) are dimensionless data provided by the accelerometry device, which are approximately proportional to the (*x*-, *y*-, and *z*-axis) directional acceleration^39^. Time *t* is discrete, which for each day *t* runs from 1 to *N*, where *N* = 24 h per day × 60 min per hour × 60 s per minute × 30 samples per second = 2,592,000 samples per day for hip-worn accelerometer data. The vector magnitude/Euclidean norm *r(t)* was computed in the hip accelerometry data as follows:

2 $$r\left( t \right) = \sqrt {x\left( t \right)^2 + y\left( t \right)^2 + z\left( t \right)^2} ,$$

To normalize the vector magnitude/Euclidean norm *r(t*) to a consistent length across both the wrist and hip accelerometry cohorts, the vector magnitude/Euclidean norm *r(t)* was reshaped to a *D* × *T* matrix ***R*** = ***R***_**dt**_ where *D* represents the total number of wear days and *T* represents collected samples per day. The average, normalized vector magnitude/Euclidean norm $\bar r\left( t \right)$ is computed as follows:

3 $$\bar r\left( t \right) = \frac{1}{D}\mathop {\sum}\limits_{d = 1}^D {R_{dt}} .$$

We then used non-overlapping 1-minute, sliding windows to extract the Euclidean norm minus one (ENMO), the counts per minute (CPM), and the vector magnitude count (VMC), both formally defined below. The ENMO was used to remove noise and gravitation effects from subminute and subsecond-level data. Letting *H* denote the number of time measurements in a one-minute sliding windo*w*, we can write ENMO as:

4 $${\mathrm{ENMO}}\left( t \right) = \frac{1}{H}\mathop {\sum}\limits_{h = 0}^{H - 1} {{\mathrm{max}}\left[ {\bar r(t + h) - 1,0} \right]} .$$

The feature CPM was further derived as:

5 $${\mathrm{CPM}}\left( t \right) = H \ast {\mathrm{ENMO}}(t)$$

Note that *H* = 60 s per sliding window × 30 samples per second = 1800 samples per sliding window for hip accelerometer data and *H* = 60 s per sliding window × 4 samples per minute = 4 samples per sliding window for wrist accelerometer data.

The VMC was used to evaluate the mean amplitude deviation in the sliding window period with size *H*, defined as:

6 $${{{{{\mathbf{VMC}}}}}}\left( {{{\boldsymbol{t}}}} \right) = \frac{1}{H}\mathop {\sum}\limits_{h = 0}^{H - 1} {\left| {\bar r(t + h) - \bar r(t)} \right|} ,$$

where *t* now varies over the minutes each day, from 1 to *N*, with *N* = 24 h per day × 60 min per hour = 1440 min per day.

#### Accelerometry activity level measures (C4 and V4)

**Fig. 5:**
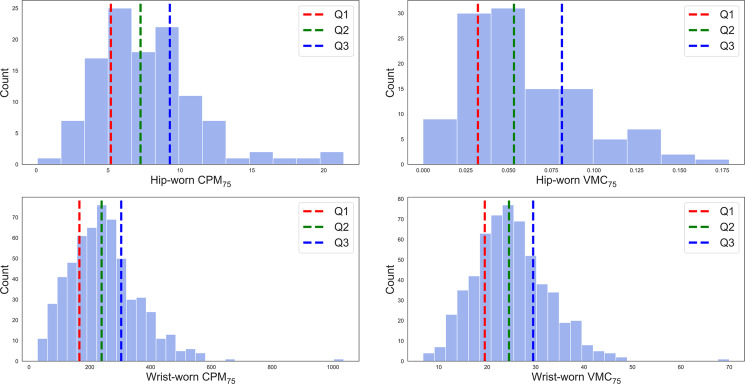
Distribution of CPM_75_/VMC_75_ over cohorts. Hip/Wrist accelerometry cohorts distribution of CPM_75_/VMC_75_. Red dashed line represents Q1 quantiles on inactive: [0, 25%]; Green dashed line represents Q2 quantiles on moderately active [25–50%]; Blue dashed line represents Q3 quantiles on active [50–75%], and above Q3 represents extremely active [75–100%].

Two categorical activity measures that we call C4 and V4 were computed. After extracting CPM and VMC measures from the accelerometer data, we generated the 75th percentile for CPM and VMC data points for each participant denoted as CPM_75_ and VMC_75_. The sample-based distribution of the CPM_75_ and the VMC_75_ were then categorized into four levels at each quartile to create a C4 and V4, respectively. Figure 5 shows the cohort-specific-based quartiles for CPM_75_ and VMC_75_ labeled as: inactive [0–25%], moderately active [25–50%], active [50–75%], and extremely active [75–100%].

#### Accelerometry pattern measures

After obtaining minute-level ENMO(*t*) and VMC(*t*), we then computed 98 statistical and harmonic features. This resulted in 105 features for those wearing the hip accelerometers (study population *N* = 115) and 104 features for those wearing the wrist accelerometers (study population *N* = 575). The number of features for the wrist- and hip-worn devices differed because the income and ethnicity/race categories in the two datasets were not identical. The specific features for ENMO(*t*) and VMC(*t*) are listed in Table 5.

**Table 5:** Statistic and harmonic features extracted from CPM (t) and VMC (t).

Statistical features
Mean and median
Standard deviation
Minimum and maximum
25th and 75th percentile
Skewness and kurtosis
Entropy
Beta distribution shape (*α,β*)
Harmonic features
Top 15 FFT^a^ Coefficients (frequency/signal)
FFT^a^ entropy
Periodogram frequency mean, Standard deviation
RMS^b^ amplitude
Periodogram frequency Kurtosis and Skewness

^a^Fast fourier transformation (FFT).

^b^Root mean squared (RMS).

**Fig. 6:**
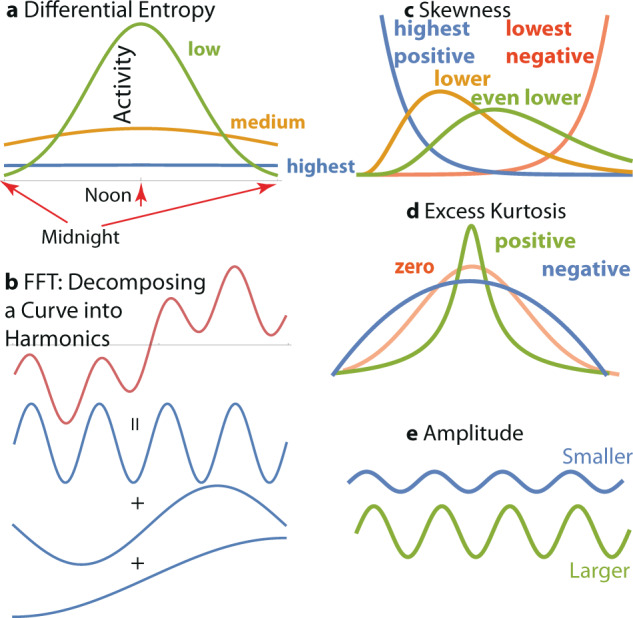
Includes animations illustrating the entropy, skewness, harmonics, kurtosis, and amplitude accelerometry features used in our analysis (we did not illustrate more standard statistics, such as mean and variance of measurements). **a** Differential entropy: Differential entropy is the highest for a uniform distribution of activity (for example, when a person stays inactive 24 h a day, so there are no bursts of activity). When a person is more active through the day and inactive at night, the entropy of activity drops, because the daytime activity exceeds the night-time activity average. **b** Fast Fourier transform (FFT): The fast Fourier transform refers to the number of harmonics that can be used to describe a curve. Any curve can be decomposed into a spectrum of harmonics. In this case, the hypothetical activity curve shown in red is the sum of 3 harmonics with nonzero amplitude: one with four cycles a day, one with a single full cycle a day, and one with a two-day cycle. In real accelerometry data, the number of accelerometry harmonics composing a 24-h circadian pattern is typically over 15 harmonics. **c** Skewness is a statistic characterizing the asymmetry of the distribution of activity; it can be applied to entire device wear time or to smaller intervals of accelerometry readings. **d** Excess kurtosis is a statistic indicating deviation of a distribution from a normal distribution. Kurtosis is zero for a normal distribution, positive for distributions with heavier (than normal) tails, such as *t*-distribution, and negative for distributions that have lighter tails, such as Beta with parameters (2,2). **e** Amplitude: The amplitude of each harmonic in an FFT reflects the distance between minimum and maximum activity values. For non-essential (noise-level) harmonics in FFT, the amplitude is close to zero.

We illustrated the meaning of the individual harmonic features in Fig. 6. While we computed a relatively large number of harmonic features, the features belong to just a few categories: differential entropy (flatness of a distribution), fast Fourier transform (revealing periodicity in activity), and statistics describing shapes of a distribution, such as mean, variance, skewness, and kurtosis.

#### Statistical analysis

First, we computed characteristics of the two cohorts: means (±standard error, SE) for continuous measures and proportions (±SE) for categorical variables. Second, to analyze the statistical significance of the covariates for distinguishing between the two classes (∆_−_, ∆_+_), we evaluated the predictive importance of each of the demographic, comorbidity, and accelerometry measure in both cohorts. Third, we built a binary classifier called CDPred to distinguish ∆_+_ from ∆_−_ in the two cohorts using XGBoost (Extreme Gradient Boosting). To evaluate the performance of the model in each cohort, we randomly chose 10% of the hip accelerometry cohort and 15% of the sample from the wrist accelerometry cohort as a hold-out sample. The CDPred hyperparameters were fine-tuned using 5-fold cross-validation, to maximize the area under the curve (AUC) score. We then reported the performance of each model in terms of predicted accuracy and AUC on a hold-out sample. The feature importance of distinguishing ∆_+_ and ∆_−_ were then listed in descending order of importance for the best-performing model in each dataset.

#### Ethics

The study was approved by the University of Chicago Institutional Review Board (IRB # 13-0443). Study participants provided written informed consent.

## Supplementary information

Supplementary Information

## Data Availability

The NSHAP data are publicly available and can be obtained from the National Archive of Computerized Data on Aging (http://www.icpsr.umich.edu/icpsrweb/NACDA/studies/34921) after completing a Data Use Agreement. The FACE Aging study data are available from one of the corresponding authors (M.H.S.) upon reasonable request and after completion of a Data Use Agreement and Institutional Review Board assessment.

## Code availability

The complete source code used in this study is available on GitHub, at https://github.com/chengjianshi/Accel1YrCog.
