# Supplementary material for: Free-living wrist and hip accelerometry forecast cognitive decline among older adults without dementia over 1- or 5-years in two distinct observational cohorts
Source: NPJ Aging. 2022 Jun 6;8(1):7. doi: 10.1038/s41514-022-00087-w (PMC9170733; doi:10.1038/s41514-022-00087-w)
Supplement: Supplementary file 1 — Supplementary Information [file 41514_2022_87_MOESM1_ESM.pdf]

Free-living wrist and hip accelerometry forecast cognitive decline among older adults without dementia over 1- or 5-years in two distinct observational cohorts: Supplementary Information

Chengjian Shi, [1,2] Niser Babiker,[2] Jacek K. Urbanek,[3] Robert L. Grossman,[2,4] Megan Huisingh-Scheetz,[2]* and Andrey Rzhetsky[2,5]*

[1]Pritzker School for Molecular Engineering, University of Chicago, Chicago, IL, 60637, USA

[2]Department of Medicine, University of Chicago, Chicago, IL, 60637, USA

[3]Department of Medicine, Johns Hopkins University, Baltimore, MD, 21287, USA

[4]Department of Computer Science, University of Chicago, Chicago, IL, 60637, USA

[5]Department of Human Genetics, University of Chicago, Chicago, IL, 60637, USA

* Co-senior authors, corresponding authors: megan.huisingh-scheetz@uchospitals.edu, andrey.rzhetsky@uchicago.edu

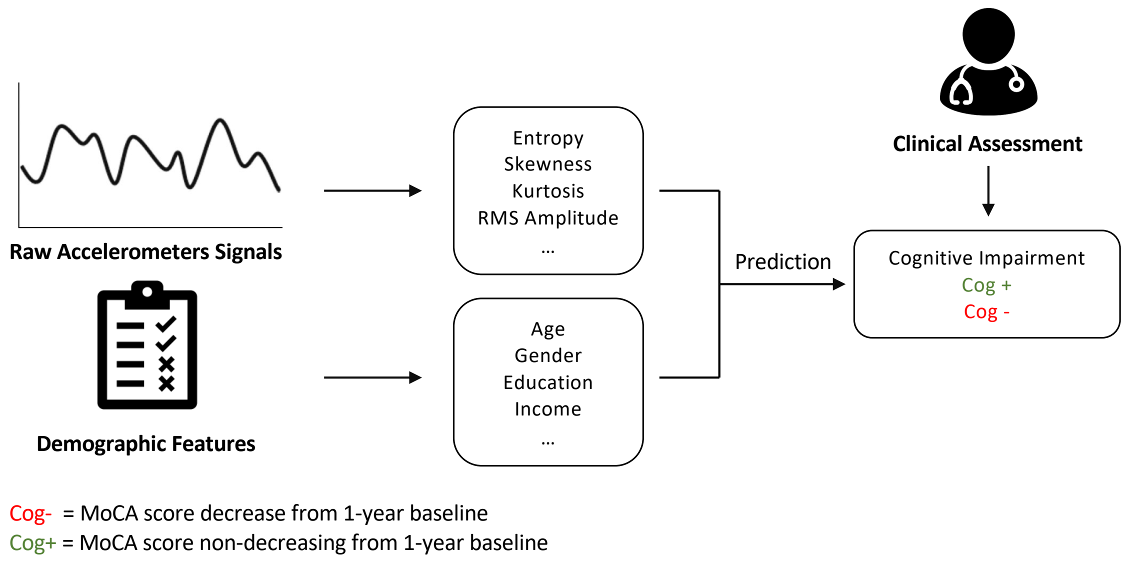
Raw Accelerometers Signals
Demographic Features
Entropy
Skewness
Kurtosis
RMS Amplitude
...
Age
Gender
Education
Income
...
Prediction
Clinical Assessment
Cognitive Impairment
Cog +
Cog -
Cog- = MoCA score decrease from 1-year baseline
Cog+ = MoCA score non-decreasing from 1-year baseline

**Features Acronym**
gender
age
ethgrp
baseline
charlson_score
CPM_mu
VMC_mu
CPM_std
VMC_std
CPM_0
VMC_0
CPM_25
VMC_25
CPM_50
VMC_50
CPM_75
VMC_75
CPM_range
VMC_range
CPM_skew
VMC_skew
CPM_kurt
VMC_kurt
CPM_beta(a)
VMC_beta(a)
CPM_beta(b)
VMC_beta(b)
CPM_entropy
VMC_entropy
CPM_PAEE
VMC_PAEE
CPM_top15fft0
VMC_top15fft0

CPM_top15fft1
VMC_top15fft1
CPM_top15fft2
VMC_top15fft2
CPM_top15fft3
VMC_top15fft3
CPM_top15fft4
VMC_top15fft4
CPM_top15fft5
VMC_top15fft5
CPM_top15fft6
VMC_top15fft6
CPM_top15fft7
VMC_top15fft7
CPM_top15fft8
VMC_top15fft8
CPM_top15fft9
VMC_top15fft9
CPM_top15fft10
VMC_top15fft10
CPM_top15fft11
VMC_top15fft11
CPM_top15fft12
VMC_top15fft12
CPM_top15fft13
VMC_top15fft13
CPM_top15fft14
VMC_top15fft14
CPM_top15freq0
VMC_top15freq0
CPM_top15freq1
VMC_top15freq1
CPM_top15freq2
VMC_top15freq2

CPM_top15freq3
VMC_top15freq3
CPM_top15freq4
VMC_top15freq4
CPM_top15freq5
VMC_top15freq5
CPM_top15freq6
VMC_top15freq6
CPM_top15freq7
VMC_top15freq7
CPM_top15freq8
VMC_top15freq8
CPM_top15freq9
VMC_top15freq9
CPM_top15freq10
VMC_top15freq10
CPM_top15freq11
VMC_top15freq11
CPM_top15freq12
VMC_top15freq12
CPM_top15freq13
VMC_top15freq13
CPM_top15freq14
VMC_top15freq14
CPM_fentropy
VMC_fentropy
CPM_psd_mu
VMC_psd_mu
CPM_psd_std
VMC_psd_std
CPM_rms_amplitude
VMC_rms_amplitude
CPM_mean_freq
VMC_mean_freq

CPM_median_freq
VMC_median_freq
educ_hs/equiv
educ_voc cert/some colle
educ_less_hs
educ_bachelors or more
income_<=$2000/month
income_$2000-3999/month
income_$4000-5999/month
income_>=$6000 /month

Description
/
/
ethnic group
MOCA Score baseline
Charlson Score
CPM mean
VMC mean
CPM standard deviation
VMC standard deviation
CPM C4 - level 1
VMC C4 - level 1
CPM C4 - Level 2
VMC C4 - level 2
CPM C4 - level 3
VMC C4 - level 3
CPM C4 - level 4
CPM C4 - level 4
range of CPM
range of VMC
CPM skewness
VMC skewness
CPM kurtosis
VMC kurtosis
CPM beta distribution alpha coeff
VMC beta distribution alpha coeff
CPM beta distribution beta coeff
VMC beta distribution beta coeff
CPM differential entropy
VMC differential entropy
CPM Physical Activity Energy Expenditure
VMC Physical Activity Energy Expenditure
CPM top 15 FFT signal - #0
VMC top 15 FFT signal - #0

CPM top 15 FFT signal - #1
VMC top 15 FFT signal - #1
CPM top 15 FFT signal - #2
VMC top 15 FFT signal - #2
CPM top 15 FFT signal - #3
VMC top 15 FFT signal - #3
CPM top 15 FFT signal - #4
VMC top 15 FFT signal - #4
CPM top 15 FFT signal - #5
VMC top 15 FFT signal - #5
CPM top 15 FFT signal - #6
VMC top 15 FFT signal - #6
CPM top 15 FFT signal - #7
VMC top 15 FFT signal - #7
CPM top 15 FFT signal - #8
VMC top 15 FFT signal - #8
CPM top 15 FFT signal - #9
VMC top 15 FFT signal - #9
CPM top 15 FFT signal - #10
VMC top 15 FFT signal - #10
CPM top 15 FFT signal - #11
VMC top 15 FFT signal - #11
CPM top 15 FFT signal - #12
VMC top 15 FFT signal - #12
CPM top 15 FFT signal - #13
VMC top 15 FFT signal - #13
CPM top 15 FFT signal - #14
VMC top 15 FFT signal - #14
CPM top 15 FFT signal frequency - #0
VMC top 15 FFT signal frequency - #0
CPM top 15 FFT signal frequency - #1
VMC top 15 FFT signal frequency - #1
CPM top 15 FFT signal frequency - #2
VMC top 15 FFT signal frequency - #2

CPM top 15 FFT signal frequency - #3
VMC top 15 FFT signal frequency - #3
CPM top 15 FFT signal frequency - #4
VMC top 15 FFT signal frequency - #4
CPM top 15 FFT signal frequency - #5
VMC top 15 FFT signal frequency - #5
CPM top 15 FFT signal frequency - #6
VMC top 15 FFT signal frequency - #6
CPM top 15 FFT signal frequency - #7
VMC top 15 FFT signal frequency - #7
CPM top 15 FFT signal frequency - #8
VMC top 15 FFT signal frequency - #8
CPM top 15 FFT signal frequency - #9
VMC top 15 FFT signal frequency - #9
CPM top 15 FFT signal frequency - #10
VMC top 15 FFT signal frequency - #10
CPM top 15 FFT signal frequency - #11
VMC top 15 FFT signal frequency - #11
CPM top 15 FFT signal frequency - #12
VMC top 15 FFT signal frequency - #12
CPM top 15 FFT signal frequency - #13
VMC top 15 FFT signal frequency - #13
CPM top 15 FFT signal frequency - #14
VMC top 15 FFT signal frequency - #14
CPM Fourier differential entropy
VMC Fourier differential entropy
CPM power spectral density mean
VMC power spectral density mean
CPM power spectral density standard deviation
VMC power spectral density standard deviation
CPM root mean squared amplitude
VMC root mean squared amplitude
CPM frequency mean
VMC frequency mean

CPM frequency median
VMC frequency median
education high school or equivalent
eduation certification / college
education less than high school
eduation bachelors or more
income less or equal to 2000 / month
income from 2000 - 3999 / month
income from 4000 - 5999 / month
income greater or equal to 6000 / month

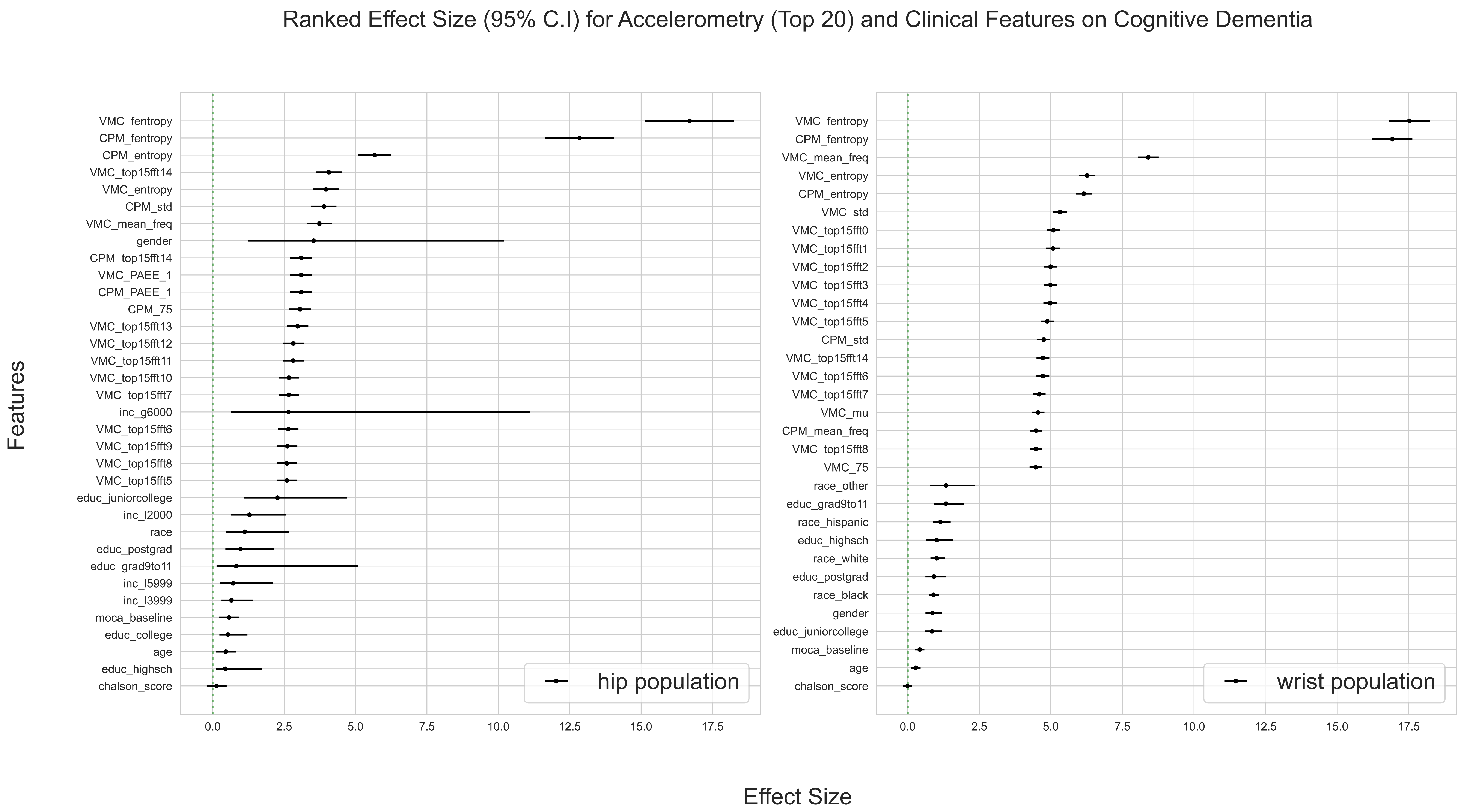
Ranked Effect Size (95% C.I) for Accelerometry (Top 20) and Clinical Features on Cognitive Dementia
Features
VMC_fentropy
CPM_fentropy
CPM_entropy
VMC_top15fft14
VMC_entropy
CPM_std
VMC_mean_freq
gender
CPM_top15fft14
VMC_PAEE_1
CPM_PAEE_1
CPM_75
VMC_top15fft13
VMC_top15fft12
VMC_top15fft11
VMC_top15fft10
VMC_top15fft7
inc_g6000
VMC_top15fft6
VMC_top15fft9
VMC_top15fft8
VMC_top15fft5
educ_juniorcollege
inc_l2000
race
educ_postgrad
educ_grad9to11
inc_l5999
inc_l3999
moca_baseline
educ_college
age
educ_highsch
chalson_score
hip population
VMC_fentropy
CPM_fentropy
VMC_mean_freq
VMC_entropy
CPM_entropy
VMC_std
VMC_top15fft0
VMC_top15fft1
VMC_top15fft2
VMC_top15fft3
VMC_top15fft4
VMC_top15fft5
CPM_std
VMC_top15fft14
VMC_top15fft6
VMC_top15fft7
VMC_mu
CPM_mean_freq
VMC_top15fft8
VMC_75
race_other
educ_grad9to11
race_hispanic
educ_highsch
race_white
educ_postgrad
race_black
gender
educ_juniorcollege
moca_baseline
age
chalson_score
wrist population
0.0
2.5
5.0
7.5
10.0
12.5
15.0
17.5
Effect Size
